# Is Parity a Risk Factor for Late Preterm Birth? Results from a Large Cohort Study

**DOI:** 10.3390/jcm13020429

**Published:** 2024-01-12

**Authors:** Lior Kashani-Ligumsky, Ran Neiger, Ella Segal, Ronnie Cohen, Miriam Lopian

**Affiliations:** 1 Department of Obstetrics and Gynecology, Mayanei Hayeshua Medical Center, Bnei Brak 51544, Israel; liorkashani@yahoo.com (L.K.-L.); cohen_ronnie@hotmail.com (R.C.); 2 Sackler School of Medicine, Tel Aviv University, Tel Aviv 69978, Israel; 3 Department of Obstetrics and Gynecology, University of South Carolina, Columbia, SC 29208, USA; ran@neiger.com

**Keywords:** preterm labor, multiparity, late preterm, nulliparity, unplanned cesarean delivery

## Abstract

Most preterm births occur in the late preterm period. While prematurity-related adverse outcomes are significantly diminished when birth occurs during this period, these infants are still at increased risk of complications. Parity affects the incidence of obstetric complications. The purpose of this study was to determine whether parity impacts the risk of spontaneous late preterm birth (SLPTB) and associated complications. A retrospective observational cohort study was conducted. Patients were divided into three study groups according to parity. The primary outcome was the rate of SLPTB in each group. Secondary outcomes were unplanned cesarean delivery (UCD), prolonged third stage of labor respiratory distress syndrome (RDS), transient tachypnea of the newborn (TTN), intraventricular hemorrhage (IVH), neonatal hypoglycemia, duration of NICU admission, neonatal death, and composite adverse neonatal outcome (CANO). Primiparas were more likely to have SLPTB, UCD, and CANO compared to multiparas (2.6% vs. 1.9% OR 1.5 [1.3–1.7] *p* < 0.01) (4.1% vs. 1.3% OR 2.7 [1.2, 5.9] *p* < 0.01) (8.5% vs. 4.2 OR 2.1 [1.3–3.5] *p* = 0.002) and grandmultiparas (2.6% vs. 1.7% OR 1.4 [1.2–1.5] *p* < 0.001) 8.5% vs. 4.4% OR 2.0 [1.1, 3.8], *p* = 0.01) but no difference in UCD compared to grandmultiparas (4.1% vs. 3.3% OR 1.2 [0.6–2.7] *p* = 0.28). Primiparas are at increased risk of SLPTB and UCD, and this is accompanied by an increased risk of adverse neonatal outcomes.

## 1. Introduction

Late preterm birth (LPTB), defined as birth between 34 + 0 and 36 + 6 weeks’ gestation, accounts for 75% of all preterm births [1]. Despite efforts to reduce the rates of these births, particularly those that are iatrogenic, their incidence has been rising steadily since the 1990s [2]. Although a large proportion of late preterm births are the consequence of maternal, fetal, and placental complications such as pre-eclampsia, pre-existing diabetes mellitus, and growth restriction, approximately 30% of these deliveries are spontaneous [3,4,5]. Even though perinatal outcomes for neonates born during this period are considerably improved compared to those born at earlier gestational ages, primarily due to a significantly reduced risk of the complications that contribute to significant neonatal morbidity and mortality, such as intra-ventricular hemorrhage and bronchopulmonary dysplasia [6], they are still worse than the outcomes of neonates born at full term [6]. In the short term, they are at increased risk of hypoglycemia, respiratory distress syndrome, jaundice, and apnea, risks that can mainly be ascribed to the structural and physiological immaturity still present at this gestational age [7]. In the long term, infants born during this period have a higher risk of neurodevelopmental impairment and psychiatric morbidity than those born at term [8]. This has been attributed to the fact that there is a high rate of neuronal development towards the end of pregnancy [9]. Indeed, one recent study evaluating the developmental and trajectories of late preterm infants indicated that over ¾ of late preterm infants had lower performance in mathematics compared to those born at term [10]; others have demonstrated a higher prevalence of attention deficit hyperactive disorder as well as autistic spectrum disorders [11] compared to children born at full term [12]. With a rising incidence of LPTB, more children are growing up with significant social and academic challenges that can place a burden on healthcare resources. Despite this, research into this cohort of patients is lacking.

Risk factors for late preterm delivery are similar to those for early preterm birth and include multiple gestations, hypertensive disorders, fetal growth restriction, maternal diabetes, and maternal obesity [13,14,15,16]. Advanced maternal age (>40 years) and high parity (>10) have been implicated as risk factors for a range of perinatal maternal and neonatal complications, including gestational diabetes mellitus, antepartum hemorrhage, fetal distress, prematurity, low birth weight, and perinatal mortality [10,17,18,19]. Data on whether and to what extent maternal parity affects the risk of late preterm birth, as well as its associated maternal and neonatal outcomes, are limited. This study aimed to evaluate the relationship between parity and the incidence of spontaneous late preterm birth as well as maternal and neonatal outcomes of birth at this point in gestation.

## 2. Methods

A retrospective observational cohort study was conducted at a single university-affiliated medical center between 2012 and 2022. The study’s primary outcome was the incidence of spontaneous late preterm birth (SLPTB) according to parity. For this analysis, demographic characteristics and the prevalence of late preterm birth were collected for all patients who had a spontaneous onset of labor after a gestational age > 23 + 6 weeks at our center. The study group was divided into three groups according to parity: Group 1—primiparas, Group 2—multiparas (parity of 1–4), and Group 3—grandmultiparas (parity ≥ 5). Demographic characteristics collected included maternal age, gestational age at delivery, parity, chronic hypertension, pregestational diabetes, a history of repeat pregnancy loss (3 or more losses), the presence of maternal anemia, defined as prenatal hemoglobin < 10.5 g/dL, gestational diabetes, and hypertensive disorders of pregnancy. Spontaneous late preterm birth was defined as spontaneous birth between 34 + 0 and 36 + 6 weeks. Patients excluded from the study group were those whose labor was indicated, i.e., underwent induction of labor or elective cesarean delivery. Pregnancies complicated by expectantly managed preterm premature rupture of membranes (PPROM), fetal demise, genetic or anatomic anomalies, and multiple gestations were also excluded from the study. The incidence of the primary outcome was compared across the three groups, and multivariate logistic regression analysis was conducted controlling for confounders such as maternal age, a history of repeat pregnancy losses, maternal anemia, pregestational diabetes, chronic hypertension, the presence of gestational diabetes, and hypertensive disorders of pregnancy. 

The secondary outcome of our study was the incidence of adverse maternal and neonatal outcomes in patients undergoing spontaneous late preterm birth. For this analysis, patients from each study group who underwent spontaneous late preterm birth were compared for the following outcomes: unplanned cesarean delivery; a prolonged third stage of labor, defined as >30 min from delivery until delivery of the placenta. Secondary neonatal outcomes included respiratory distress syndrome (RDS), transient tachypnea of the newborn (TTN), and neonatal hypoglycemia, defined as glucose < 25 mg/dL in the first 4 h of life or <35 mg/dL at 4–24 h of life, duration of neonatal intensive care unit (NICU) admission, neonatal death, and a composite adverse neonatal outcome which was comprised of a neonatal APGAR score < 7 at 5 min, umbilical artery pH < 7.1 and NICU admission > 14 days. Data were collected from a computerized patient database.

Statistical analyses were performed using IBM SPSS statistics version 26.0, Armonk, NY, USA: IBM Corp. Continuous variables were analyzed using ANOVA or *t*-test. Non-continuous variables were analyzed using the Chi-square test. Multivariate logistic regression analysis was performed for the primary outcome. Results were considered statistically significant when *p* < 0.01. The Institutional Review Board approved the study. Approval number is MHMC-0025-21.

## 3. Results

Between 2012 and 2022, there were 115,552 deliveries at our institution, and 80,956 (70.1%) occurred after a spontaneous onset of labor. Of those, 60 (0.1%) were very early preterm births, born between 24 + 0 and 27 + 6 weeks; 396 (0.5%) were early preterm births, born between 28 + 0 and 33 + 6 weeks; 1595 (2.0%) were late preterm births; and 78,905 (97%) were spontaneous term births, born after 37 + 0 weeks (Table 1).

Demographic characteristics of patients undergoing spontaneous delivery during the study period are presented in Table 1 and Demographic characteristics of patients who had a SLPTB are presented in Table 2.

With regards to the primary outcome, after adjusting for confounders, primiparas (Group 1) were significantly more likely to have a SLPTB compared to multiparas (Group 2) (2.6% vs. 1.9% OR 1.5, [1.3–1.8] *p* < 0.001) and grandmultiparas (Group 3) (2.6% vs. 1.7% OR 1.4 [1.2–1.5] *p* < 0.01). There was no significant difference in the risk of SLPTB in multiparas (Group 2) compared to grandmultiparas (Group 3) (1.9% vs. 1.7%, OR 1.1, [1.0–1.3], *p* = 0.04 (Table 1.).

Secondary outcomes were compared across the three groups for the 1595 (2.0%) patients who underwent SLPTB (Table 3 and Table 4). With regards to maternal outcomes, primiparas and grandmultiparas were also more likely to experience unplanned cesarean delivery compared to multiparous patients (4.1% vs. 1.3% OR 2.7 [1.2, 5.9], *p* < 0.01) and (3.3% vs. 1.3% OR 0.4 [0.2–0.9] *p* = 0.01. There was no difference in the risk of cesarean delivery between primiparas and grandmultiparas (4.1% vs. 3.3% OR 1.2 0.6–2.7 *p* = 0.28). The most common indication for cesarean delivery in all three groups was malpresentation, which occurred in 6/14 primiparas (42.8%), 8/12 multiparas (66.7%), and 10/12 grandmultiparas (83.3%). There were no significant differences between the groups in the rate of prolonged third stage of labor (Table 3 and Table 4).

With regard to secondary neonatal outcomes, there were no significant differences between the groups in the rates of RDS (10.3%, 11.5%, and 10.5%), TTN (2.9%, 4.1%, and 2.2%), hypoglycemia (1.8%, 1.0% and 1.4%), and mean duration of NICU admission (11.9, 9.6, and 11.7 days) in Group 1, 2, and 3, respectively. Our study had no neonatal death cases (Table 3). Primiparas were more likely to experience the CANO compared with multiparas (8.5% vs. 4.2 OR 2.1 CI [1.3–3.5], *p* = 0.002) and grandmultiparous women (8.5% vs. 4.4% OR 2.0 CI [1.1, 3.8], *p* = 0.01). There were no differences in the composite CANO between multiparas and grandmultiparas (4.2% vs. 4.4% OR 1.1 [0.6–2.2] *p* = 0.3) (Table 3).

A total of 42,419 (52%) patients had more than one delivery during the ten-year study period. These patients were included in the statistical analysis since this finding is to be expected in a study of long duration. Nevertheless, once these patients were removed, there were 38,338 (48%) patients who only delivered once during the study period. The results of the primary outcome did not significantly differ when restricting the analysis to patients with only one delivery. Primiparas had a higher risk of SLPTB compared to multiparas (2.6% vs. 1.9% OR 1.3 95% CI 1.1–1.5 *p* < 0.001) and grandmultiparas (2.6% vs. 1.8% OR 1.4 95% CI 1.2–1.8 *p* < 0.001). There were no significant differences in the risk of SLPTB between multiparas and grandmultiparas (1.9% vs. 1.8% OR 1.1 95% CI 0.9–1.9 *p* = 0.2).

## 4. Discussion

The results of our study demonstrate that primiparous patients are at increased risk of spontaneous late preterm birth as well as a combined adverse neonatal outcome compared to multiparas and grandmultiparas. We also show that primiparas had an increased risk of unplanned cesarean delivery during this period compared to multiparas but not grandmultiparas.

The rate of preterm delivery remains higher than 10% in many regions of the world. This rate is affected by geographic and ethnic differences; for example, in the United States, it was 10% in 2022, while in Nordic and Baltic countries, it was 5–7% [20,21,22]. Indeed, the etiology of preterm birth is complex and multifactorial [23]. Risk factors implicated in preterm birth include advanced maternal age [24], high body mass index (BMI) [25], history of prior preterm delivery [26], multiple gestations [27], maternal infection [28], as well as uterine [29] or fetal anomalies [14]. There is a lack of data as well as controversy in the literature regarding the effect of parity on maternal and obstetric outcomes. Earlier studies have reported higher risks of adverse outcomes, particularly of postpartum hemorrhage and malpresentation [30] in these women, while more recent studies demonstrate that grandmultiparity poses no additional maternal risks, particularly in high-resource settings [20,31].

Concerning the effect of parity on the risk of preterm birth, several studies report that primiparas are at increased risk of preterm birth compared to multiparous patients [32,33,34,35]. A recent large retrospective cohort study by Koullali et al. [32] of over 30,327 spontaneous preterm births reported that primiparous patients were almost twice as likely than parous patients to undergo spontaneous preterm birth (OR 1.95 95% CI 1.89–2.0). When analyzing the effect of parity on birth at different gestational ages, they demonstrated that the impact of primiparas is increased risk of spontaneous preterm birth persisted at <28 (aOR 2.02), <32 (aOR 2.15), and 34–37 (aOR 1.85) weeks and it persisted even when controlling for confounders such as age, ethnicity, socioeconomic status, and prior preterm birth. Primiparity had the most significant effect on early births < 32 weeks compared to those 34–37 weeks. Interestingly, the reference group for this study was patients with a parity of 1. When analyzing patients with a parity > 1, the risk of spontaneous late preterm birth increased with increasing parity until a parity of 4 across all gestational age groups (<28, <32, 34–37, <37 weeks). Despite this, nulliparous patients still had the highest risk of SLPTB of all the parity groups for PTB at all gestational ages except for births at <28 weeks. For these extremely preterm births, patients with a parity of 4 had a higher risk of SPTB compared to nulliparas (aOR 2.44 vs. 2.02), suggesting that while primiparity seems to be a risk factor for preterm birth overall, the risk starts to increase again with increasing parity > 1. Furthermore, the patients with a parity > 4 are at the highest risk for extreme preterm births, which are those associated with the highest rates of adverse neonatal outcomes. These results suggest potentially different mechanisms involved in extreme preterm births compared to later preterm births. We also demonstrate a similar increased risk of SLPTB in primiparous patients with aOR of 1.4 and 1.5 compared to 1.85 reported by Koullali. However, with regard to the effect of grandmultiparity, Koullali demonstrated an increasing risk of preterm birth with increasing parity > 1, particularly for extreme preterm birth [32]; results from this study as well as a large systemic review of over 41 studies, did not demonstrate that increasing parity was associated with an increased risk of preterm birth both at early and later gestational ages [30]. Differences in our study populations could explain the discrepancies in our findings, our study had a higher proportion of multiparous patients, and unlike Koullali, we also evaluated the outcomes of grandmultiparous patients (parity > 5). Indeed, this group comprised over a quarter of our study cohort. In addition, we did not separately analyze the impact of each parity group as a continuum but rather compared three groups; primiparas, multiparas, and grandmultiparas.

The role parity plays on this risk of preterm birth is complex due to the various confounding factors closely associated with parity such as age [24], co-morbidities [28], and BMI [25], all of which are also independently associated with preterm birth. The mechanism by which nulliparity increases the risk of spontaneous preterm birth is yet to be elucidated. One possible theory may be related to variations in vaginal microbiota. Indeed there are several lines of evidence suggesting that the vaginal microbiota are influenced by parity and gestational age [36,37,38], and several studies have demonstrated that this microbiota can influence the risk of several pregnancy outcomes, most notably spontaneous preterm birth [39]. More studies evaluating the mechanisms by which parity influence preterm birth are needed to fully understand this association.

Aside from this current study and that by Koulalli [32], to our knowledge no other studies have evaluated the effect of parity on late spontaneous preterm birth. Although neonates born in the late preterm have improved outcomes compared to those born at earlier gestational ages [6], they constitute the majority of preterm births [1] and therefore their contribution to overall neonatal outcomes is not insignificant. Ascertaining risk factors underlying these births is critical for several reasons as patients at risk might benefit from counselling as well as targeted prevention strategies to reduce this risk. Furthermore, the results of the Antenatal Betamethasone for Women at Risk for Late Preterm Delivery (ALPS) Trial demonstrated a significant benefit in reducing adverse neonatal outcomes in singletons born in the late preterm period who received antenatal corticosteroids [39] and both the American College of Obstetrics and Gynecology as well as the Society of Maternal and Fetal Medicine recommend considering their use in patients at risk of delivering in the late preterm period [40,41]. A better understanding of who is at risk for late preterm birth will help direct this treatment, and the results of this and the Koullali study both suggest that consideration for lowering the threshold for administering steroids in primiparas who present with threatened preterm labor compared to multiparas.

Our study also demonstrated that primiparas having a spontaneous late preterm birth were at increased risk of the composite adverse neonatal outcome compared to multiparas (8.5% vs. 4.2 OR 2.1 CI [1.3–3.5], *p* = 0.002) and grandmultiparas (8.5% vs. 4.4% OR 2.0 CI [1.1, 3.8], *p* = 0.01). There were no differences between groups in the risk of RDS, TTN, hypoglycemia, IVH and neonatal death, and average NICU admission duration. This effect was driven primarily by higher rates of low APGAR scores at 5 min and prolonged NICU admission in primiparas. It is possible that this could be the result of a higher rate of unplanned cesarean delivery in primiparas or the lower mean birthweight seen in these patients, both factors that are known to contribute to poor short-term neonatal outcomes [42,43] and further suggests that these patients are higher risk and may benefit from antennal steroid treatment.

## 5. Strengths and Limitations

The strength of this study is the large study population of over 80,000 spontaneous preterm births, with a significant proportion of the cohort defined as multiparas (57.7%) and grandmultiparas (26%). This allowed us to evaluate better the effect of parity, an essential factor and one that is often understudied, on the risk of SLPTB. The limitations of this study are its retrospective nature and the lack of data regarding the effect of other confounding variables such as smoking, obesity, socioeconomic status, a history of preterm birth, and the rates of antenatal corticosteroid administration, which might have influenced outcomes.

## 6. Conclusions

Compared to multiparous patients, primiparas are at increased risk for spontaneous delivery in the late preterm period. Primiparas are also at increased risk of unplanned cesarean delivery and adverse neonatal outcomes when delivering during the late preterm period. This information might help in stratifying the risk for spontaneous late preterm birth, the consequences of which can impact patient counseling, particularly in primiparas for whom this might be their first birth. Furthermore, risk stratification can aid decision-making in patients with suspected preterm labor during this period, particularly regarding the administration of antenatal corticosteroids.

## Figures and Tables

**Table 1:** Demographic characteristics in patients who had a spontaneous birth at >23 + 6 weeks.

	Nuliparas	Multiparas	Grandmultiparas	*p*-Value
Number	13,097	46,791	21,068	
Maternal age (mean, years)	23.3 ± 3.3	27.8 ± 4.0	35.5 ± 3.8	<0.01
Maternal age < 25 (n, %)	10,135 (77.4%)	10,415 (22.2%)	4 (0.02%)	<0.01
Maternal age 25–34 (n, %)	2764 (21.1%)	33,212 (70.1%)	8986 (42.6%)	<0.01
Maternal age > 35 (n, %)	198 (1.5%)	3164 (6.7%)	12,078 (57.3%)	<0.01
Gestational age at delivery (mean, weeks + days)	39 + 2 ± 1.6	39.5 ± 1.3	39.3 ± 1.3	<0.01
Neonatal birth weight (mean, grams)	3223 ± 437	3357 ± 431.7	3443 ± 437	<0.01
Chronic Hypertension (n, %)	62 (0.47%)	125 (0.27%)	59 (0.28%)	<0.01
Anemia (Hb < 10.5) (n, %)	211 (1.6%)	1547 (3.3%)	767 (3.6%)	<0.01
Pregestational diabetes (n, %)	83 (0.63%)	222 (0.47%)	104 (0.49%)	0.07
Repeat pregnancy loss (n, %)	53 (0.4%)	868 (1.85%)	1464 (6.94%)	<0.01
Previous cesarean delivery (n, %)	n/a	4010 (8.6%)	2939 (13.9%)	<0.01
Hypertensive disorders of pregnancy (n, %)	90 (0.68%)	152 (0.32%)	94 (0.44%)	<0.01
Gestational diabetes (n, %)	862 (6.6%)	2341 (5.0%)	1257 (5.9%)	<0.01
VEPTB (24–28 weeks) N = (%)	24 (0.18%)	30 (0.1%)	6 (0.03%)	<0.01
EPTB (28–34 weeks) N = (%)	75 (0.6%)	162 (0.3%)	149 (0.2%)	<0.01
Late PTB n = 1595, (2.0%)	339 (2.6%)	895 (1.9%)	361 (1.7%)	<0.01
Term births	12,659 (96.7%)	45,704 (97.7%)	20,552 (97.6%)	<0.01

Continuous variables reported as means ± standard deviation; n/a: not applicable. VEPTB: very early preterm birth—between 24 + 0–27 + 6 weeks. EPTB: early preterm birth—between 28 + 0–33 + 6 weeks. Late PTB: preterm birth between 34 + 0–36 + 6 weeks.

**Table 2:** Demographic characteristics of patients with spontaneous late preterm births.

	Primiparas	Multiparas	Grandmultiparas	*p*-Value
Number	339	895	361	
Maternal age (years)	23.3 +/− 3.8	27.5 +/− 4.8)	35.7 (+/−4.0)	<0.01
Maternal age < 25	264 (77.9%)	251 (28.0%)	0	<0.01
Maternal age 25–34	65 (19.2%)	573 (64%)	144 (39.9%)	<0.01
Maternal age > 35	10 (2.9%)	71 (7.9%)	217 (60.1%)	<0.01
Maternal Anemia (Hb < 10 g/dL)	7 (2.1%)	39 (4.3%)	26 (7.0%)	<0.01
Chronic hypertension (n, %)	4 (1.18%)	2 (0.22%)	0	0.06
Pregestational diabetes (n, %)	3 (0.88%)	11 (1.2%)	0	0.2
Repeat pregnancy loss (n, %)	2 (0.59%)	20 (2.2%)	33 (9.1%)	<0.01
Previous cesarean delivery (n, %)	n/a	16 (1.8%)	16 (4.4%)	<0.01
Hypertensive disorders of pregnancy (n, %)	4 (1.18%)	15 (1.67%)	6 (1.6%)	0.8
Gestational diabetes (n, %)	29 (8.5%)	60 (6.7%)	24 (6.6%)	0.4
Gestational age at delivery (weeks + days)	35 + 3 ± 0.7	35 + 4 ± 0.69	35 + 4 ± 0.69	<0.01
Neonatal birth weight (grams)	2546 (+/−382.4)	2667 (+/−384.4)	2713 (+/−416.9)	<0.01

Continuous variables reported as means ± standard deviation; n/a: not applicable.

**Table 3:** Prevalence of maternal and neonatal outcomes in patients who had a spontaneous late preterm birth.

	Primiparas N = 339	Multiparas N = 895	Grandmultiparas N = 361
Cesarean delivery (n, %)	14 (4.1%)	12 (1.3%)	12 (3.3%)
Prolonged third stage (n, %)	8 (2.3%)	17 (1.9%)	9 (2.5%)
Neonatal RDS (n, %)	35 (10.3%)	103 (11.5%)	38 (10.5%)
TTN (n, %)	10 (2.9%)	37 (4.1%)	8 (2.2%)
Neonatal hypoglycemia (n, %)	6 (1.8%)	9 (1.0%)	5 (1.4%)
Neonatal death (n, %)	0 (0%)	0 (0%)	0 (0%)
Average NICU admission duration (days)	11.9	9.6	11.7
Umbilical artery PH < 7.1 (n, %)	2 (0.6%)	1 (0.1%)	1 (0.3%)
Apgar < 7 at 5 min (n, %)	7 (2.1%)	7 (0.8%)	6 (1.7%)
NICU admission > 14 days (n, %)	20 (5.9%)	30 (3.3%)	9 (2.5%)
Combined adverse neonatal outcome (n, %)	29 (8.5%)	38 (4.2%)	16 (4.4%)

RDS—Respiratory Distress Syndrome, TTN—Transient Tachypnea of the Newborn, IVH—Intraventricular hemorrhage, NICU—Neonatal Intensive Care Unit.

**Table 4:** Maternal and Neonatal outcomes in patients with spontaneous late preterm births according to parity.

	Primiparas vs. Multiparas	Primiparas vs. Grandmultiparas	Multiparas vs. Grandmultiparas
Spontaneous late preterm birth	aOR 1.5	aOR 1.4	aOR 1.1
	[1.3–1.8]	[1.2–1.5]	[1–1.3]
	*p* < 0.01	*p* < 0.01	*p* = 0.04
Cesarean delivery	OR 2.7	OR 1.2	OR 0.4
	[1.2–5.9]	[0.6–2.7]	[0.2–0.9]
	*p* < 0.01	*p* = 0.28	*p* = 0.01
Prolonged third stage	OR 1.2	OR 0.9	OR 0.8
	[0.5–2.9]	[0.4–2.5]	[0.3–1.7]
	*p* = 0.3	*p* = 0.4	*p* = 0.2
Neonatal RDS			
	OR 0.88	OR 0.98	OR 1.1
	[0.2–1.4]	[0.6–1.6]	[0.7–1.6]
	*p* = 0.5	*p* = 0.4	*p* = 0.5
TTN			
	OR 0.7	OR 1.3	OR 1.9
	[0.3–1.4]	[0.5–3.4]	[0.5–3.4]
	*p* = 0.1	*p* = 0.2	*p* = 0.05
Neonatal hypoglycemia			
	OR 1.7	OR 1.3	OR 0.7
	[0.6–5.0]	[0.4–4.2]	[0.2–2.2]
	*p* = 0.1	*p* = 0.3	*p* = 0.2
Neonatal death			
	N/A	N/A	N/A
Average NICU admission duration (days)			
	0.09	0.9	0.3
Umbilical artery PH < 7.1	OR 5.3	OR 2.1	OR 0.4
	[0.5–58.7]	[0.2–23.7]	[0.03–6.5]
	*p* = 0.08	*p* = 0.2	*p* = 0.2
APGAR < 7 at 5 min	OR 2.7	OR 1.2	OR 0.4
	[0.9–7.7]	[0.4–3.8]	[0.16–1.4]
	*p* = 0.03	*p* = 0.3	*p* = 0.08
NICU admission > 14 days	OR 1.8	OR 2.4	OR 1.3
	[1.0, 3.2]	[1.1–5.5]	[0.6–2.9]
	*p* = 0.02	*p* = 0.01	*p* = 0.2
Combined adverse neonatal outcome			
	2.1	2	1.1
	[1.3–3.5]	[1.1–3.8]	[0.6–2.2]
	*p* = 0.002	*p* = 0.01	*p* = 0.3

RDS—respiratory distress syndrome, TTN—transient tachypnea of the newborn, NICU—neonatal intensive care unit, N/A—Not applicable, OR—odds ratio, aOR—adjusted odds ratio.

## Data Availability

Data are available in the article.
